# Operationalizing Chronic Inflammation: An Endotype-to-Care Framework for Precision and Equity

**DOI:** 10.3390/clinpract15120233

**Published:** 2025-12-10

**Authors:** Maria E. Ramos-Nino

**Affiliations:** Department of Microbiology, Immunology, and Pharmacology, St. George’s University School of Medicine, St. George P.O. Box 7, Grenada; mramosni@sgu.edu

**Keywords:** chronic inflammation, endotypes, biomarkers, precision medicine, equity, immunometabolism, inflammasome, microbiome, senescence

## Abstract

**Background/Objectives:** Chronic inflammation arises from self-reinforcing immune–metabolic circuits encompassing pattern-recognition signaling, inflammasome activation, cytokine networks, immunometabolic reprogramming, barrier–microbiome disruption, cellular senescence, and neuro–immune–endocrine crosstalk. This review synthesizes these mechanistic axes across diseases and introduces an operational endotype-to-care framework designed to translate mechanistic insights into precision-based, scalable, and equitable interventions. **Methods:** A narrative, mechanism-focused review was performed, integrating recent literature on immune–metabolic circuits, including pattern-recognition receptors, inflammasome pathways, cytokine modules, metabolic reprogramming, barrier–microbiome dynamics, senescence, and neuro–immune–endocrine signaling. Validated, low-cost screening biomarkers (hs-CRP, NLR, fibrinogen) were mapped to phenotype-guided endotyping panels and corresponding therapeutic modules, with explicit monitoring targets. **Results:** We present a stepwise, pragmatic pathway progressing from broad inflammatory screening to phenotype-specific endotyping (e.g., IL-6/TNF for metaflammation; ISG/IFN for autoimmunity; IL-23/17 for neutrophilic disease; IL-1β/NLRP3 or urate for crystal-driven inflammation; permeability markers for barrier–dysbiosis). Each module is paired with targeted interventions and prespecified treat-to-target outcomes: for example, achieving a reduction in hs-CRP (e.g., ~40%) within 8–12 weeks is used here as a pragmatic operational benchmark rather than a validated clinical threshold. Where feasible, cytokine and multi-omic panels further refine classification and prognostication. A tiered implementation model (essential, expanded, comprehensive) ensures adaptability and equity across clinical resource levels. **Conclusions:** Distinct from prior narrative reviews, this framework defines numeric triage thresholds, minimal endotype panels, and objective monitoring criteria that make chronic inflammation management operationalizable in real-world settings. It embeds principles of precision, equity, and stewardship, supporting iterative, evidence-driven implementation across diverse healthcare environments.

## 1. Introduction

Inflammation is an evolutionarily conserved host defense program with the dual purposes of eliminating danger signals and orchestrating repair [1]. It is normally self-limited by clearance of pathogens or wound healing and resolution. When the inciting trigger is unresolved or homeostatic brakes are lost, the response becomes chronic and self-sustaining [1,2]. There are reciprocal positive feedback loops between immune cells and stromal compartments that reinforce the response as well as integration with the nervous system and the metabolic state [2,3]. Clinically, chronic inflammation manifests along a spectrum of conditions from low-grade ‘inflammaging’ that can be measured by blood C-reactive protein (CRP) and cytokines, to tissue-destructive autoimmunity and fibrosis [1,2,4]. Identifying the nodes where inflammation converts from a transient process to chronic is therefore key to primary prevention as well as treatment [4,5,6]. Distinct from prior umbrella reviews, this work specifies numeric triage thresholds, minimal endotype panels, and paired treat-to-target markers that enable deployment in resource-constrained settings.

### 1.1. Cellular and Molecular Mechanisms

#### 1.1.1. Pattern Recognition and Danger Sensing

Innate pattern-recognition receptors (PRRs) include Toll-like receptors (TLRs), RIG-I–like receptors, cGAS–STING, and NOD-like receptors (NLRs) [2,7,8]. Chronic inflammatory disease is often linked to constant exposure to metabolic (cholesterol crystals, advanced glycation end-products [AGEs], extracellular ATP, oxidized LDL, uric acid) or sterile danger ligands that maintain tonic PRR signaling. These receptors, particularly TLRs and NLRs, tend to skew to the transcriptional programs associated with persistent NF-κB and interferon regulatory factor (IRF) activation with chronic cytokine production [4,7,9,10,11].

#### 1.1.2. Inflammasomes and Pyroptosis

The NLRP3 inflammasome, which integrates signals from ion flux, mitochondrial stress, and lysosomal damage, can activate caspase-1 to process IL-1β/IL-18 cytokines and induce a lytic form of cell death called pyroptosis [4,12,13]. NLRP3 is primed by NF-κB–dependent transcription after engagement of TLRs or other receptors such as LPS-sensing CD14. Metabolic disease and gout are examples where crystalline ligands directly prime and activate NLRP3, while in neurodegeneration, mitochondrial DNA released by damaged mitochondria and misfolded proteins are important persistent stimuli [4,12]. Activation of non-canonical inflammasomes via caspase 4/5/11 expands the repertoire of potential targets in chronic inflammatory conditions [12,14,15,16,17].

#### 1.1.3. Cytokine and Chemokine Networks

A number of pro-inflammatory modules (TNF, IL-1, IL-6, GM CSF, IL-17/23 axis) and type I/II interferons (IFNs) interact with stromal-derived factors (TGF β, VEGF) and chemokines (CCL2, CXCL8) to perpetuate leukocyte recruitment, tissue remodeling, and angiogenesis [1,7,18]. Chronicity in many cases reflects an imbalance between these modules and specific pro-resolving mediators (lipoxins, resolvins, protectins, maresins) [1,3,12]. In vivo, each of these cytokines can drive immunometabolic reprogramming and secretion of one another, leading to broad amplification loops [19,20,21].

#### 1.1.4. Immunometabolism and Mitochondrial Dysfunction

Activated innate myeloid cells shift to aerobic glycolysis and the pentose phosphate pathway, leading to an accumulation of TCA cycle intermediates, most notably succinate and citrate, which stabilize HIF 1α and fuel prostaglandin and nitric oxide (NO) synthesis [1,2,4]. Mitochondrial reactive oxygen species (ROS) and defective mitophagy are also important sources of persistent DAMPs in chronic inflammation [2,4]. In lymphocytes, metabolic checkpoints govern cell fate between effector and regulatory phenotypes, and either nutrient excess or nutrient deprivation can tip this balance in the wrong direction [2,7,22].

#### 1.1.5. Epigenetic Reprogramming and Trained Immunity

Sustained stimuli induce chromatin accessibility and histone mark changes in innate immune progenitors and macrophages, a form of plasticity called trained immunity [2,7]. This has beneficial roles in fighting infections but can be maladaptive if the stimuli (Western diet, hyperglycemia, air pollutants) maintain a state of hyper-responsiveness and drive chronic inflammation [2,23,24].

#### 1.1.6. Barrier Failure and Microbiome Dysbiosis

Tissue barrier dysfunction increases translocation of microbial products and metabolites across epithelial and endothelial linings [1,7]. Dysbiosis, a loss of short-chain fatty acid (SCFA) producers and expansion of ‘pathobionts’, modulates TLR/NOD receptor signaling, Treg development, and bile acid and tryptophan metabolic pathways and can propagate systemic low-grade inflammation [1,7,25,26,27,28].

#### 1.1.7. Senescence, SASP, and Inflammaging

An accumulation of senescent cells that secrete a pro-inflammatory senescence-associated secretory phenotype (SASP) can amplify chronic inflammation, and both senescent immune and stromal cells contribute to impaired resolution and tissue repair [4,12,29,30,31].

#### 1.1.8. Neuro–Immune–Endocrine Crosstalk

The hypothalamic–pituitary–adrenal (HPA) axis, sympathetic tone, and vagal anti-inflammatory pathways all bidirectionally modulate cytokine expression [3,32]. Chronic stress, sleep loss, and circadian disruption also rewire these circuits to lower the threshold for persistent inflammation [1,3,33,34].

### 1.2. From Mechanism to Disease

#### 1.2.1. Cardiometabolic Disorders

Atherosclerosis, obesity, and type 2 diabetes are prototypical diseases of sterile low-grade inflammation. The myeloid activation state maintained by lipid antigens and hyperglycemia drives IL-1/6/TNF loop signaling, and resident macrophages in adipose tissue and hepatocytes are key to remodeling metabolic set points [1,4,18,35]. Actionable signals: hs-CRP, NLR, IL-6, TNF; metabolic markers (HOMA-IR, triglycerides). Module-directed options include lifestyle/weight loss, metformin/GLP-1RA/SGLT2i, and, selectively, TNF or IL-6R blockade per guidelines and comorbidity.

#### 1.2.2. Cancer

Chronic inflammation is a key driver of oncogenesis via tissue damage, DNA damage, angiogenesis, and immunoediting. Macrophages and neutrophils in the tumor microenvironment shape a cytokine milieu (IL-6, IL-1β, TNF, CXCL8) that supports tumor growth and metastasis and counteracts antitumor immunity [1,36,37].

#### 1.2.3. Neurodegeneration

Microglial priming, disrupted proteostasis, and mitochondrial injury are drivers of chronic neuroinflammation in Alzheimer’s, Parkinson’s, and related disorders, with NLRP3 inflammasome activation and defective resolution contributing to synaptic and mitochondrial dysfunction [4,12,13,38].

#### 1.2.4. Autoimmunity and Autoinflammation

Breakdown of self-tolerance and aberrant cytokine axes (IL-17/23, type I IFN) lead to sustained tissue damage in diseases such as rheumatoid arthritis, inflammatory bowel disease, psoriasis, and systemic lupus erythematosus [1,32,39]. Monogenic autoinflammatory syndromes demonstrate a causal role in inflammasome pathways [12]. Psoriasis and psoriatic arthritis (PsA) are IL-23 → IL-17A/F-driven disorders with neutrophil-skewed chemokines (CXCL1/8) and stromal activation. Biomarkers: IL-17-linked chemokines, clinical PASI/DAPSA. Therapies: IL-23 (guselkumab/risankizumab) or IL-17A/F (secukinumab/bimekizumab) inhibitors with clear treat-to-target outcomes.

#### 1.2.5. Fibrosis and Organ Failure

Chronic inflammatory signaling engages TGF β, platelet-derived growth factor (PDGF), and pathways leading to myofibroblast activation and extracellular matrix (ECM) deposition. This leads to architectural distortion and organ failure in lung, liver, kidney, and heart [1,18].

#### 1.2.6. Post-Infectious Sequelae

After acute infections, persistence of viral antigens, tissue damage, or immune imprinting can drive ongoing chronic inflammation and dysautonomia, as may be seen in certain post-viral syndromes [1,4].

### 1.3. Biomarkers and Endotyping

Low-cost single markers such as CRP, serum amyloid A (SAA), and select cytokines are imperfect but useful for stratification in at-risk individuals or early disease. Multiplex panels that include inflammatory tone (CRP, SAA), cytokine modules (IL-6, TNF, IL-1β, IL-17/23), major immune cell subsets (neutrophil/lymphocyte ratio, monocyte subsets, Treg/Th17), metabolic stress markers (ketone bodies, acylcarnitines), and circulating or tissue injury markers (troponins, fibrosis biomarkers) can be assembled for robust risk stratification [1,2,4]. Integration of multiple ‘omics’ (transcriptome, proteome, metabolome, microbiome) and machine-learning classifiers can enable derivation of actionable endotypes that can predict risk and therapeutic response [4,6,29,40].

### 1.4. Therapeutic Strategies

#### 1.4.1. Lifestyle and Environmental Interventions

Diet patterns that emphasize whole, minimally processed foods and fiber, regular physical activity, restorative sleep, stress reduction, and avoidance of environmental exposures (air pollution, smoking) have general efficacy at lowering the inflammatory set point [1,3]. Weight loss and improved glycemic control specifically attenuate IL-6/TNF signaling and improve metabolic–immune coupling [4].

#### 1.4.2. Anti-Cytokine and Immune-Modulating Therapies

Biologics targeting TNF, IL-6R, IL-1β, IL-17/23, type I IFN signaling, and GM CSF have transformed the care of many inflammatory conditions, and small-molecule JAK inhibitors that broadly modulate cytokine pathways have further expanded our armamentarium [18,32]. S1P modulators, costimulation blockers, and B-cell–depleting agents also show value in lymphocyte-driven diseases [1].

#### 1.4.3. Inflammasome and Danger-Signaling Inhibitors

Direct NLRP3 inhibitors, caspase-1 inhibitors, and purinergic (P2X7) antagonists are newer options for selected inflammasome-driven disease [4,12,14,15,16,17], and upstream TLR antagonists and cGAS–STING pathway modulators are in development for treating sterile inflammation and autoimmunity [7]. Clinical adoption remains investigational; current evidence is limited to early-phase trials with uncertain durability and safety profiles across indications.

#### 1.4.4. Immunometabolic Reprogramming

Metformin, GLP-1 receptor agonists, and AMPK activators improve metabolic–immune coupling [1,4,22,41,42], and mTOR, HIF 1α, and glycolysis inhibitors as well as targeted antioxidant strategies (mitochondria-directed ROS scavengers; Nrf2 activators) can modulate maladaptive myeloid activation [2].

#### 1.4.5. Resolution Pharmacology

Specialized pro-resolving mediators (SPMs) such as resolvins, protectins, maresins, and 15 LOX–derived lipoxins promote efferocytosis, barrier repair, and a return to homeostasis without broad immunosuppression [1,12,43,44,45]. Human evidence remains early and heterogeneous; durability and optimal dosing strategies are unresolved.

#### 1.4.6. Senolytics and Senomorphics

Senolytic drugs that selectively eliminate senescent cells or senomorphic drugs that selectively suppress the SASP have promise for addressing inflammaging and fibrosis [4,6,12,31], but safety, durability, and off-target effects are active areas of investigation. Senolytics/senomorphics are promising but remain investigational; current human data are limited and indication specific.

#### 1.4.7. Neuromodulation and Mind–Body Adjuncts

Vagus nerve stimulation and behavioral interventions that can increase parasympathetic tone can reduce cytokine output and are useful as adjuncts in some conditions [3,32].

## 2. Materials and Methods

### 2.1. Study Design and Objective

This narrative, mechanism-informed review synthesizes available evidence to create an actionable endotype-to-care framework for chronic inflammation to inform precision prevention and treatment, spanning from mechanisms to actionable endotypes, clinical biomarkers, therapeutic modules, and monitorable outcomes. The framework is designed for use in the general adult population at-risk for chronic inflammation, as well as adults with established chronic inflammatory diseases, including cardiometabolic, autoimmune, autoinflammatory, barrier–microbiome, and inflammaging-related conditions. In addition, the framework is meant to be transdiagnostic across all levels of prevention: primary prevention (population biomarker screening and lifestyle/exposome strategies), secondary prevention (individuals with early inflammatory or metabolic abnormalities), and tertiary prevention (patients with established chronic inflammatory disease requiring targeted therapeutics and longitudinal treat-to-target monitoring) No meta-analysis was conducted.

### 2.2. Protocol and Methodology

The review protocol (sources, search terms, inclusion/exclusion criteria, data extraction and evidence grading) was established a priori, but was not registered, due to the non-systematic nature of the review. Methodological quality criteria followed those of the Society for Academic Nursing’s Guidelines for Systematic and Non-Systematic Reviews (SANRA) for narrative reviews. The PRISMA statement was not applicable.

### 2.3. Literature Sources and Search Dates

This review is narrative and non-systematic in design; the literature search was broad, flexible, and iterative, not protocolized. The search strings were wide-ranging, encompassing mechanistic, clinical, metabolic, and endotype-related concepts, and were refined over time until the point of thematic saturation was reached. Iterative cycles of literature searching were conducted in PubMed/MEDLINE, Embase, and Web of Science, and supplemental searching was performed in Google Scholar primarily for forward citation chasing and was limited to the first 150–200 results of each search query to minimize noise from relevance-ranking algorithms. Reference lists from a set of sentinel mechanistic papers, high-quality systematic reviews, and clinical guidelines were hand-searched for additional articles.

Sentinel clinical guidelines (EULAR/ACR, AGA/ACG, ACC/AHA, GINA/GOLD), clinical trial registries, and the reference lists of included articles were also searched for additional evidence. The last search date of the electronic databases was 12 November 2025.

#### Search Terms

Mechanistic, clinical, and translational concepts were combined using Boolean operators. Mechanistic terms included innate immune–sensing pathways (pattern-recognition receptors, inflammasomes, cGAS–STING), immunometabolism, dysbiosis, senescence-associated secretory phenotype (SASP), and inflammatory mediators (IL-1, IL-6, TNF, IL-17/23, IFNs, GM-CSF). Clinical and translational terms included endotypes, biomarkers, and treat-to-target strategies. Therapeutic and intervention-related terms included biologics, JAK inhibitors, GLP-1 agonists, senolytics, specialized pro-resolving mediators, and inhibitors targeting NLRP3 or cGAS–STING pathways. Equity and implementation concepts included exposome, digital biomarkers, and circadian biology.

### 2.4. Eligibility Criteria and Study Selection

Mechanistic, translational, and clinical studies of all types; randomized and quasi-experimental trials; high-quality observational studies; systematic reviews/meta-analyses; expert and guideline statements; and Phase I–III reports were eligible for inclusion, with human studies prioritized and animal/mechanistic studies used to address evidence gaps/interpret relevance to human biology. Untriangulated preprints, case reports, and non-English articles not synthesized in the body of the manuscript were excluded.

### 2.5. Data Extraction and Endotype-to-Care Framework Construction

Key variables abstracted: endotype class, associated biomarker(s)/threshold(s), module-specific interventions, targets/outcome metrics for monitoring, safety and equity considerations. Biomarker selection was driven by 3 criteria: (1) having been endorsed in key consensus guidelines or regulatory frameworks, (2) having robust mechanistic or clinical effect-size evidence in the chronic inflammatory biology of interest, and (3) being technically feasible in diverse resource settings. Thresholds were harmonized across centers using guideline-based cut-points where available. In the absence of consensus cut-points, and where high platform/assay heterogeneity was expected (e.g., in the case of cytokines and interferon-stimulated gene signatures), we interpret these biomarkers as “direction-of-change” classifiers rather than absolute diagnostic values. Repeat testing and trend-based monitoring will be a focus to minimize misclassification. Evidence supporting endotype-based interventions was triangulated/graded in 3 tiers:

A = Guideline-supported/approved;

B = Strong clinical/mechanistic but limited regulatory approval;

C = Emerging/experimental.

Review algorithmically translates screening steps (e.g., hs-CRP, NLR, fibrinogen) into endotype identification, module-targeted therapy selection, and treat-to-target outcome benchmarks (e.g., hs-CRP lowered ≥40% at 8–12 weeks, disease activity measures, urate-to-target).

To avoid overinterpreting the available evidence, biomarkers and thresholds were cross-referenced to levels of evidence (A–C). Level A biomarkers are those supported by guidelines or that have regulatory approval (e.g., hs-CRP, IL-6, TNF), level B are those with strong clinical or mechanistic evidence but with limited or no approvals, and level C are those that are experimental or emerging (e.g., multi-omic classifiers, senescence signatures). Thresholds lacking harmonized clinical validation were to be interpreted as directional trends and not absolute diagnostic cut-points.

### 2.6. Operationalizing the Endotype-to-Care Framework and Equity in Implementation

Clinical pathways and algorithmic steps were adapted for resource-rich and -limited environments, from minimum/essential markers and lifestyle-first approaches at the population level to expanded biomarker panels and omics profiling in more comprehensive clinical/research settings. Elements of decentralized, task-shifted care delivery (e.g., dried blood spot collection, digital adherence reminders/monitoring, remote treatment targets/outcome checks) were also integrated for purposes of equity and feasibility.

The endotype-to-care framework was derived by integrating evidence maps across mechanistic axes (cytokine profiles, metabolic pathways, ISG signatures), biomarker feasibility across resource settings, and principles of treat-to-target design borrowed from rheumatology, cardiometabolic medicine, and implementation science. The final framework reflects the convergence of three factors: (1) mechanistic coherence, (2) feasibility of measurement and intervention, and (3) alignment with modifiable pathways of disease progression.

### 2.7. Quality Assurance and Reporting

The study selection was based on the highest level of evidence from available mechanistic, translational, and clinical research. As an exercise in methodological transparency, biomarker heterogeneity was accounted for with explicit language: guideline-derived cut-points were used if validated; otherwise, relative change and trends over time were emphasized over single absolute values to reduce the impact of inter-assay variability. When different cytokine or multi-omic platforms were used, results were considered based on the directionality and reproducibility of findings rather than numeric equivalency. Conflicts between studies were noted where relevant, and evidence gaps, uncertainties, and assay variability were acknowledged. All reporting was per best-practice guidance for narrative reviews.

The operational thresholds and multi-omic approaches discussed in this review are meant to be conceptual frameworks that should be further tested in future investigations. Their integration into this review was included as an indication of an evolving area of study and is not a current standard of care.

## 3. Results

### 3.1. Implementation: From Endotype to Care Pathway

**Figure 1:**
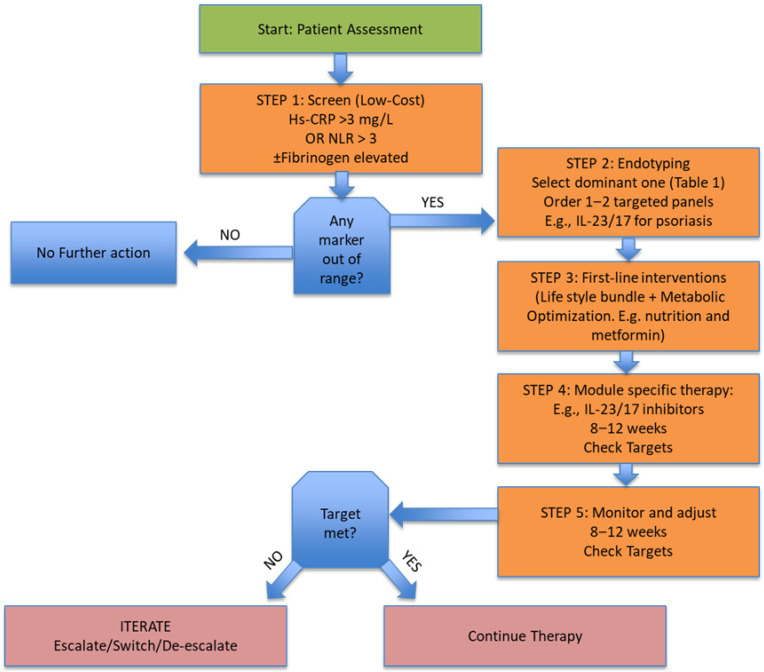
Endotype-to-Care Pathway for Chronic Inflammation. Algorithm illustrating a stepwise approach from low-cost inflammatory screening to targeted intervention. Step 1: population or clinic screening with hs-CRP, NLR, and/or fibrinogen identifies individuals with elevated inflammatory tone (hs-CRP > 3 mg/L or NLR > 3). Step 2: mechanistic endotyping uses targeted cytokine panels selected by phenotype, metabolic (IL-6/TNF), autoimmune (ISG/IFN), neutrophilic (IL-23/17), crystal-driven (IL-1β/NLRP3), or barrier–dysbiosis (permeability markers). Step 3: first-line lifestyle and metabolic optimization precede Step 4: module-specific therapy (e.g., TNF/IL-6R/IL-1 blockade, JAK or IL-23/17 inhibition). Step 5: iterative monitoring at 8–12 weeks applies treat-to-target logic (hs-CRP ↓ ≥ 40% or module-specific marker response) with escalation, maintenance, or de-escalation as indicated. The pathway emphasizes practicality, precision, and equity across resource settings. Elements such as treat-to-target reductions, multi-omic classifiers, and detailed cytokine panels are included as conceptual operational tools and should not be interpreted as universally validated or clinically mandated.

We offer a pragmatic, iterative framework to translate mechanistic understanding into actionable concepts that inform clinical and population practice aligned with the primary, secondary, and tertiary prevention applications defined in Section 2.1; however, several components, particularly multi-omic classifiers and certain cytokine-based endotyping panels, should be viewed as emerging proposals rather than validated clinical standards (Figure 1).

**Step 1:** Screening Using Inexpensive Biomarkers. Population-based screening and risk stratification can employ validated inexpensive markers such as high-sensitivity C-reactive protein (hs-CRP), serum amyloid A (SAA), fibrinogen, or complete blood count–derived indices (neutrophil-to-lymphocyte ratio [NLR], platelet-to-lymphocyte ratio [PLR]), among others. High levels of CRP (>3 mg/L) and NLR (>3) have been consistently associated with cardiometabolic, oncologic, and frailty outcomes [1,46,47,48]. If out of range proceed to step 2.

**Step 2:** Mechanistic Endotyping (Table 1). People with elevated inflammatory tone may be further defined by targeted cytokine panels and, when possible, actionable multi-omic classifiers integrated across metabolic, epigenomic, and microbiome data. Because evidence strength varies across these tools, cytokine panels and multi-omic classifiers should be interpreted according to their evidence tier (A–C), with several elements still considered exploratory rather than validated clinical standards.

Panel selection should be guided by the predominant clinical and metabolic context. Individuals with metabolic features such as central adiposity or insulin resistance may be evaluated using IL-6/TNF panel testing, whereas those with autoimmune features, such as photosensitivity, anti-dsDNA positivity, or low complement, may be tested using an ISG/Type I interferon panel. Neutrophilic skin or joint disease favors assessment with IL-17/23 panels, while crystal arthropathies or recurrent inflammatory flares warrant IL-1β, urate, and NLRP3 inflammasome profiling. In cases characterized by gastrointestinal symptoms or evidence of metabolic endotoxemia, evaluation of intestinal permeability markers and stool microbiome profiles is recommended. Where assay variability or platform heterogeneity is substantial (e.g., cytokine assays), biomarkers should be used primarily as direction-of-change indicators rather than strict diagnostic thresholds.

These targeted assays help refine age-associated and metabolic inflammatory endotypes [2,49], and multi-omics integration has shown particular utility for distinguishing disease subtypes [4,12,18,50]. However, multi-omic classifiers remain an emerging adjunct and should be applied cautiously until additional validation studies are available.

**Step 3:** Stepwise Treatment Approach. Treatment should first emphasize lifestyle and environmental interventions that reduce the inflammatory baseline and lower the set point, including nutrition, physical activity, stress reduction, sleep, and avoidance of pollution and other inflammatory stimuli. Nutritional and metabolic factors are key modulators of cardiometabolic and immune risk and remain major drivers of inflammation [51,52]. Neuroimmune signaling and vagal anti-inflammatory pathways also contribute to inflammatory tone and are targets for behavioral interventions [32].

**Step 4:** Precision, Evidence-Based, Mechanistically Targeted Therapeutics. In persons whose endotyping or clinical phenotyping points to specific inflammatory pathways, targeted therapeutics can be applied to that inflammatory “module.” These include, for example, IL-6 receptor or TNF-blocking agents, JAK inhibitors, inflammasome antagonists, and senolytics, among others, each with mechanistic and clinical rationales in chronic inflammatory disease [4,12,18,50]. Before initiating biologics or JAK inhibitors, standard infection, malignancy, and cardiovascular risk screening should be performed in accordance with clinical guidelines, and antimicrobial and steroid stewardship must be documented as part of safety oversight.

**Step 5:** Iterative Monitoring and Treatment Optimization (8–12 weeks). Follow-up and treat-to-target can be accomplished with repeat screening using the inexpensive markers above (hs-CRP, NLR, fibrinogen), among others, and supported by cytokine or metabolite panels when indicated in complex or mixed phenotypes. Emerging digital health measures and real-time biometric analytics can also be leveraged for dynamic therapy titration [53].

**Monitoring rule:** Review at 8–12 weeks; if the target is not achieved and adherence is adequate, escalate within the same module before switching modules; de-escalate after two consecutive target achievements.

This five-tiered strategy maps onto three foundational principles of precision, equity, and stewardship (Figure 1). It aims to ensure that at a minimum, inflammatory burden is assessed at the population level in low-resource settings while also providing a framework to try to provide evidence-based, mechanism-based treatments for high-risk individuals.

**Table 1:** Chronic Inflammation Endotypes → Biomarkers → Candidate Therapies → Monitoring.

Endotype	Key Biomarkers/Drivers	Example Therapies	Monitoring Targets	Evidence Tier
Type 2 (T2-high) [54,55,56,57]	IL-4/IL-5/IL-13; eosinophils; IgE; FeNO; periostin	Anti-IL-5/IL-5R; anti-IL-4Rα; anti-TSLP; ICS	↓ Eosinophils (<150–300/µL), ↓ FeNO, fewer exacerbations	A
Type 2–low (neutrophilic/mixed) [58,59,60]	Th1/Th17 signals; sputum/blood neutrophils; low IgE/eos	Macrolides; anti-alarmins; IL-17/23 blockade (select cases)	↓ Neutrophils; ↓ CRP/IL-6 ↓	B
IL-23/Th17 (Type 3) [61,62,63,64]	IL-23 → IL-17A/F; CXCL1/8; neutrophil-skewed transcriptomics	Anti-IL-23; anti-IL-17A/F	↓ PASI/DAPSA; ↓ IL-17-linked chemokines	A
Type I IFN [65,66,67]	ISG score; IFN-α/β proteins; low complement	IFNAR blockade; B-cell–directed agents	↓ ISG score; fewer flares; steroid-sparing	A
IL-1β/Inflammasome [68,69,70]	NLRP3 activation; IL-1β/IL-18; urate crystals	IL-1 blockade; emerging NLRP3/P2X7 inhibitors	↓ IL-1β/CRP; ↓ flares; urate-to-target	A/C
GM-CSF-driven [71,72,73]	High GM-CSF; inflammatory monocytes/macrophages	Anti-GM-CSF/GM-CSF-R	↓ Joint counts; ↓ CRP/SAA; ↓ synovitis (imaging) ↓	B
TNF/IL-6 a (metaflammation) [2,18,74]	hs-CRP/SAA high; IL-6/TNF loops; insulin resistance	Anti-TNF; IL-6R blockade; GLP-1RA/SGLT2i/metformin	↓ hs-CRP ≥ 40%; ↓ IL-6 ≥ 30%; ↓ HOMA-IR; metabolic improvement	A/B
Barrier–dysbiosis/endotoxemia [75,76,77,78]	LBP/LPS; EndoCab; dysbiosis; bile acid/Trp shifts	Diet pattern therapy; fiber/pre/probiotics; bile acid modulators	↓ LBP/EndoCab; ↑ stool diversity; ↓ CRP/IL-6	B/C
Senescence/SASP [79,80,81]	p16; SASP cytokines; frailty markers	Senolytics/senomorphics; SPM analogs; exercise	↓ IL-6/CRP; ↑ function (grip, gait)	C
Fibrotic TGF-β–dominant [82,83,84]	TGF-β/SMAD; PRO-C3; fibrosis imaging	Approved anti-fibrotics; investigational TGF-β modulators	Stabilize/improve fibrosis; ↓ ECM biomarkers	A/B
Complement/immune-complex [85,86,87]	Low C3/C4; C3a/C5a; autoantibodies; IC deposition	C5/C3 inhibitors; B-cell–directed therapy	↓ Complement fragments; ↓ organ activity; ↓ flares	A

Abbreviations (alphabetized): CBC, complete blood count; CXCL, C-X-C motif chemokine ligand; DAPSA, Disease Activity in Psoriatic Arthritis; ECM, extracellular matrix; EndoCab, Endotoxin Core Antibody; FeNO, fractional exhaled nitric oxide; FVC, forced vital capacity; GLP-1RA, GLP-1 receptor agonist; GM-CSF, granulocyte–macrophage colony-stimulating factor; HOMA-IR, Homeostatic Model Assessment of Insulin Resistance; IC, immune complex; ICS, inhaled corticosteroid; ISG, interferon-stimulated gene; LBP, LPS-binding protein; LPS, lipopolysaccharide; PASI, Psoriasis Area and Severity Index; p16INK4a, cyclin-dependent kinase inhibitor p16; PRO-C3, N-terminal pro-peptide of type III collagen; SAA, serum amyloid A; SASP, senescence-associated secretory phenotype; SMAD, SMAD family signaling proteins; SGLT2i, sodium–glucose cotransporter-2 inhibitor; SPM, specialized pro-resolving mediator; Trp, tryptophan; TSLP, thymic stromal lymphopoietin. Interpretation of Evidence Grading. A: Approved/Guideline-Supported: Established efficacy; incorporated in major guidelines or regulatory approval (e.g., TNF, IL-6R, IL-5, IL-23/17, IL-1, IFNAR, organ-specific anti-fibrotics, complement inhibitors). B: Clinical Signal: Promising phase II–III or strong mechanistic/observational evidence but limited approvals (e.g., GM-CSF blockade, metabolic add-ons, microbiome or fibrosis modulators). C: Emerging/Experimental: Preclinical, early-phase, or pilot translational data (e.g., NLRP3 inhibitors, TLR4 antagonists, senolytics, SPM analogs). ↓: Reduced, ↑: Increased.

### 3.2. Tiered Implementation by Resource Level

Implementation of the endotype-to-care pathway can be adapted to diverse resource environments through a tiered approach that preserves clinical validity while maximizing feasibility.

**Tier 1 (Essential Tier):** Population and primary-care settings may rely on low-cost markers such as hs-CRP, NLR, and fibrinogen to stratify inflammatory risk, paired with lifestyle optimization and metformin where appropriate. Follow-up should use a 12-week treat-to-target loop based on repeat hs-CRP or NLR.

**Tier 2 (Expanded Tier):** Centers with moderate laboratory capacity can add a single cytokine panel (for example, IL-6/TNF or IL-1β) to refine mechanistic attribution and, where available, incorporate metabolic co-targets such as fasting insulin or HOMA-IR. GLP-1 receptor agonists may be considered as metabolic–anti-inflammatory adjuncts.

**Tier 3 (Comprehensive Tier):** Specialized or research facilities can deploy multiplex cytokine panels and selected multi-omic assays (transcriptomic, metabolomic, or microbiome profiling) to enable individualized endotyping and therapeutic precision, including access to biologics, targeted small-molecule inhibitors, or clinical trials.

**Task-Shifting and Remote Monitoring**

To support equitable delivery, elements of screening and follow-up can be decentralized through task-shifting and digital health integration. Community health workers can facilitate collection of dried blood spots for hs-CRP or related biomarkers, enabling remote or population-level surveillance without venipuncture infrastructure. SMS-based adherence prompts, and symptom check-ins can reinforce behavioral and pharmacologic adherence between visits. Group consultations every three months may optimize clinician efficiency, peer support, and monitoring continuity in resource-constrained settings. These decentralized strategies lower access barriers while maintaining fidelity to the 8–12-week treat-to-target framework.

### 3.3. Worked Case Vignette (Operational Example)

This vignette is an illustrative, entirely synthetic example created solely to demonstrate how the endotype-to-care framework operates in practice. It does not represent real patient data and does not constitute human-subject research.

A middle-aged person with obesity/T2D; hs-CRP 6.2 mg/L, NLR 3.4, triglycerides high. Endotype panel: IL-6 high, TNF modest; ISG negative; IL-23/17 negative.

**Plan:** lifestyle bundle + GLP-1RA; consider IL-6R/TNF inhibition only if guideline-supported and comorbid inflammatory disease present.

**12-week monitoring:** hs-CRP ↓ 52%, weight −7%, HOMA-IR improved → continue bundle; reassess quarterly.

## 4. Evidence Gaps and Controversies

Current challenges in operationalizing inflammatory endotyping include several unresolved scientific and implementation issues. Overlapping endotypes and mixed phenotypes often blur mechanistic boundaries, limiting the precision of single axis targeting. Lack of assay harmonization across cytokine and interferon-stimulated gene (ISG) platforms, together with pre-analytic variability related to time of sampling, diet, and microbiome composition, underscores the need for standardized protocols and cross-platform calibration. Generalizability across ancestries, sexes, and environmental contexts remains insufficiently validated, requiring ancestry-specific and geographically diverse studies. Equity gaps persist in access to diagnostics and therapies, particularly the differential availability of biologics versus metabolic interventions in resource-limited settings. Finally, long-term safety, durability, and off-target effects of emerging agents such as senolytics, specialized pro-resolving mediator (SPM) analogs, and inflammasome or cGAS–STING inhibitors warrant extended follow-up and post-marketing surveillance.

The purposeful design of the framework included both evidence-based (Tier A) and investigative (Tiers B and C) aspects. The suggested practical steps including endotyping panels, multi-omic classifiers, and treat-to-target cut-offs are all currently potential, evolving strategies that should not be considered clinical guidelines.

## 5. Future Directions

Future research should move beyond correlative frameworks to causal perturbation multi-omics capable of dissecting directionality in immune–metabolic circuits. Advances in integrated transcriptomic, metabolomic, proteomic, and epigenomic profiling can now enable temporal mapping of disease progression and therapy response in vivo [26,27,28]. Longitudinal, interventional studies using CRISPR-based perturbations, single-cell spatial multi-omics, and systems modeling are essential to define the hierarchical nodes that convert transient inflammation into chronic pathology.

A second priority is the integration of “inflammatory clocks”, quantitative signatures derived from inflammatory cytokine networks, immune cell phenotypes, and DNA-methylation or proteomic surrogates of biological age [6,29,30]. These markers can provide standardized measures of “inflammatory age” and could be incorporated into risk stratification algorithms within the proposed endotype-to-care pathway. Embedding such clocks into multi-omic risk models may refine the precision of early detection, treatment titration, and evaluation of intervention durability.

In parallel, senescence-targeted interventions, including senolytics, senomorphics, and metabolic or immune modulators, are rapidly transitioning from preclinical validation to early human trials [31]. Understanding the tissue-specific immune–stromal interactions that drive SASP persistence will guide safer and more selective strategies to restore resolution and tissue homeostasis.

Finally, future frameworks must address environmental, microbial, and social determinants of chronic inflammation. Harmonizing exposome and digital biomarkers, captured via wearable devices, circadian and behavioral metrics, and geospatial pollution data—can contextualize molecular signals with real-world exposures [86,87,88,89]. Integrating these data streams through equitable digital infrastructures will enable precision public health approaches that extend beyond high-resource environments.

Collectively, these priorities, causal perturbation multi-omics [26,27,28], inflammatory clock integration [6,29,30], senescence-targeted interventions [29], and exposome/digital biomarker harmonization [88,89,90,91], define the next translational frontier for operationalizing chronic inflammation endotyping into scalable, equitable prevention and care strategies.

## 6. Conclusions

Chronic inflammation arises from self-reinforcing immune–metabolic circuits that are embedded in tissue ecology. Mechanistic endotyping and targeted multi-domain intervention have the potential to turn this growing liability into actionable precision care. Coupling biological discoveries to implementation science, as well as attention to equity and long-term safety, will be critical for success.

## Data Availability

All data is presented in the text.

## Abbreviations

The following abbreviations are used in this manuscript:

ACG	American College of Gastroenterology
ACR	American College of Rheumatology
AGEs	Advanced glycation end-products
AMPK	AMP-activated protein kinase
ATP	Adenosine triphosphate
BCR	B-cell receptor
CBC	Complete blood count
cGAS	Cyclic GMP–AMP synthase
CRISPR	Clustered regularly interspaced short palindromic repeats
CRP	C-reactive protein
CXCL	C-X-C motif chemokine ligand
DAPSA	Disease Activity in Psoriatic Arthritis
DAMPs	Damage-associated molecular patterns
dsDNA	Double-stranded DNA
ECM	Extracellular matrix
ELISA	Enzyme-linked immunosorbent assay
EndoCab	Endotoxin core antibody
EULAR	European Alliance of Associations for Rheumatology
FeNO	Fractional exhaled nitric oxide
FVC	Forced vital capacity
GLP-1RA	Glucagon-like peptide-1 receptor agonist
GM-CSF	Granulocyte–macrophage colony-stimulating factor
HIF-1α	Hypoxia-inducible factor 1 alpha
HOMA-IR	Homeostatic Model Assessment of Insulin Resistance
HPA	Hypothalamic–pituitary–adrenal
hs-CRP	High-sensitivity C-reactive protein
IC	Immune complex
ICS	Inhaled corticosteroid
IFN	Interferon
IFNAR	Type I interferon receptor
IgE	Immunoglobulin E
IL	Interleukin
IRF	Interferon regulatory factor
ISG	Interferon-stimulated gene
JAK	Janus kinase
LBP	LPS-binding protein
LDL	Low-density lipoprotein
LOX	Lipoxygenase
LPS	Lipopolysaccharide
mAb	Monoclonal antibody
mTOR	Mechanistic target of rapamycin
MTX	Methotrexate
NADPH	Nicotinamide adenine dinucleotide phosphate
NF-κB	Nuclear factor kappa-light-chain-enhancer of activated B cells
NLR	Neutrophil-to-lymphocyte ratio
NLRs	NOD-like receptors
NLRP3	NOD-, LRR-, and pyrin domain-containing protein 3
NO	Nitric oxide
PASI	Psoriasis Area and Severity Index
PDGF	Platelet-derived growth factor
PLR	Platelet-to-lymphocyte ratio
P2X7	Purinergic receptor P2X7
PRR	Pattern-recognition receptor
PRO-C3	Procollagen type III N-terminal propeptide
qPCR	Quantitative polymerase chain reaction
ROS	Reactive oxygen species
SAA	Serum amyloid A
SASP	Senescence-associated secretory phenotype
SCFA	Short-chain fatty acids
SGLT2i	Sodium–glucose cotransporter-2 inhibitor
SMAD	Mothers against decapentaplegic homolog
SPM	Specialized pro-resolving mediator
SPMs	Specialized pro-resolving mediators
STING	Stimulator of interferon genes
T2D	Type 2 diabetes
TCA cycle	Tricarboxylic acid cycle
TGF-β	Transforming growth factor beta
Th17	T helper 17
TLA	Three-letter acronym
TLR	Toll-like receptor
TNF	Tumor necrosis factor
Treg	Regulatory T cell
Trp	Tryptophane
TSLP	Thymic stromal lymphopoietin
VEGF	Vascular endothelial growth factor
